# Supplementary material for: clipplotr—a comparative visualization and analysis tool for CLIP data
Source: RNA. 2023 Jun;29(6):715–23. doi: 10.1261/rna.079326.122 (PMC10187674; doi:10.1261/rna.079326.122)
Supplement: Supplemental Material [file supp_29_6_715__DC1.html]

clipplotr—a comparative visualization and analysis tool for CLIP data — Supplemental Material 

# *clipplotr*—a comparative visualization and analysis tool for CLIP data

## Supplemental Material

- Supplemental\_Fig\_S1.pdf
- Supplemental\_Fig\_S2.pdf
- Supplemental\_Figure\_Legends.docx
