# Supplementary material for: clipplotr—a comparative visualization and analysis tool for CLIP data
Source: RNA. 2023 Jun;29(6):715–23. doi: 10.1261/rna.079326.122 (PMC10187674; doi:10.1261/rna.079326.122)
Supplement: Supplemental Material [file supp_079326.122_Supplemental_Fig_S1.pdf]

Supplemental Figure S1

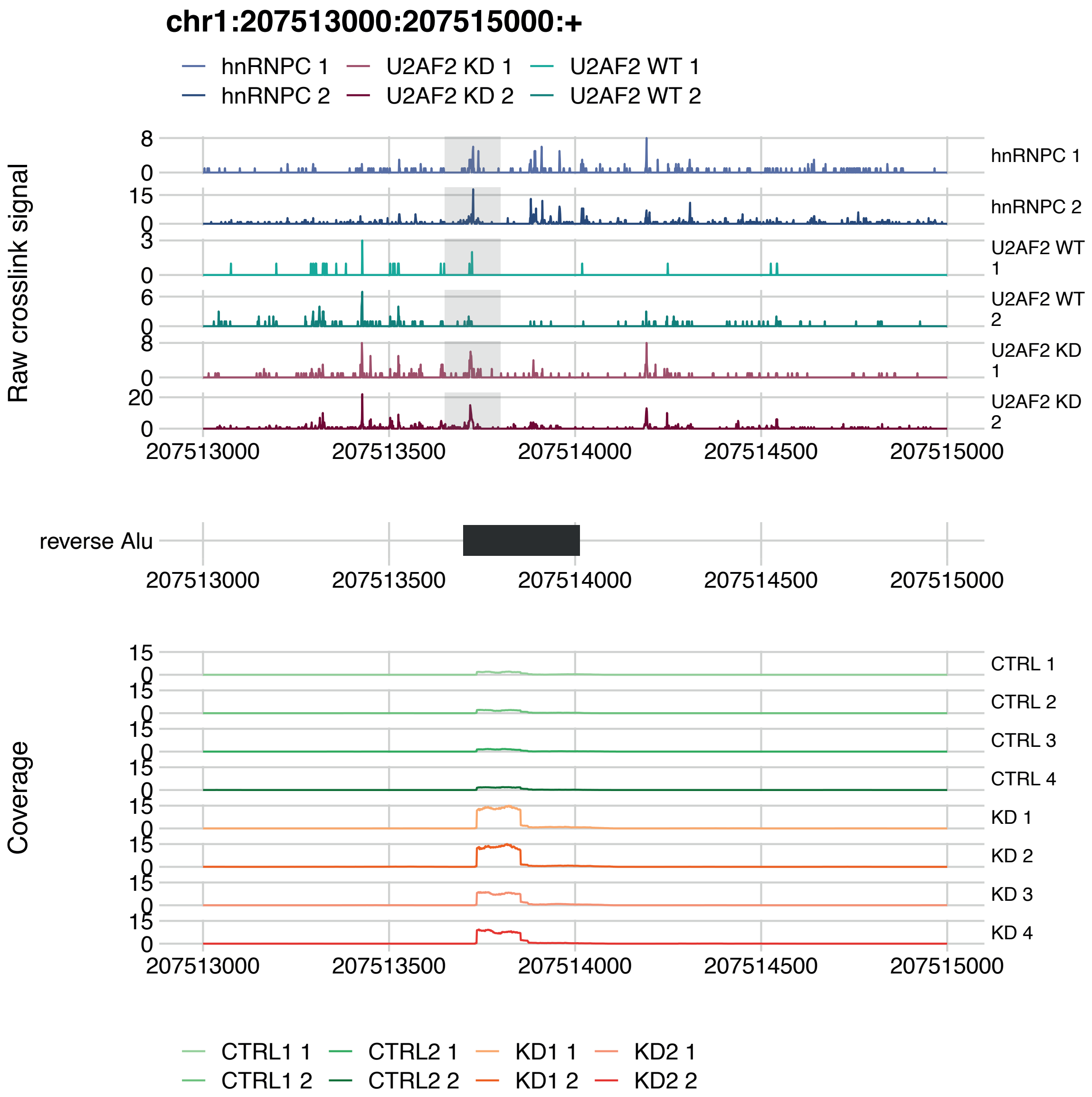

Supplemental Fig. S1. Related to Fig. 1. A reproduction using *clipplotr* of the original visualisation approach from Zarnack et al. (2013) of the CLIP and RNA-seq signal at the CD55 *Alu* exonisation locus.
