# Supplementary figures and images for: clipplotr—a comparative visualization and analysis tool for CLIP data
Source: RNA. 2023 Jun;29(6):715–23. doi: 10.1261/rna.079326.122 (PMC10187674; doi:10.1261/rna.079326.122)

**A**

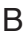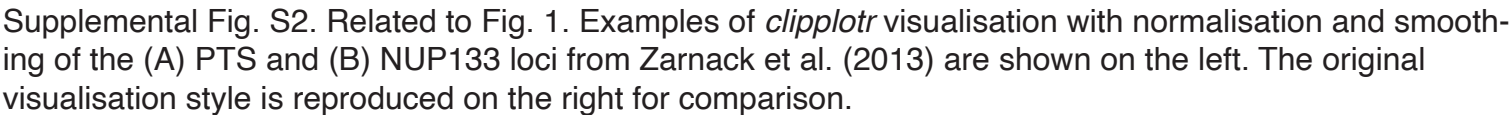

Supplement: Supplemental Material [file supp_079326.122_Supplemental_Fig_S2.pdf]
