# Supplementary material for: clipplotr—a comparative visualization and analysis tool for CLIP data
Source: RNA. 2023 Jun;29(6):715–23. doi: 10.1261/rna.079326.122 (PMC10187674; doi:10.1261/rna.079326.122)
Supplement: Supplemental Material [file supp_079326.122_Supplemental_Figure_Legends.docx]

### Supplementary Figure S1

Related to Fig. 1. A reproduction using *clipplotr* of the original visualisation approach from Zarnack et al. (2013) of the CLIP and RNA-seq signal at the CD55 *Alu* exonisation locus.

### Supplementary Figure S2

Related to Fig. 1. Examples of *clipplotr* visualisation with normalisation and smoothing of the (A) PTS and (B) NUP133 loci from Zarnack et al. (2013) are shown on the left. The original visualisation style is reproduced on the right for comparison.
